# Supplementary material for: Modeling glioblastoma heterogeneity as a dynamic network of cell states
Source: Mol Syst Biol. 2021 Sep 16;17(9):e10105. doi: 10.15252/msb.202010105 (PMC8444284; doi:10.15252/msb.202010105)
Supplement: Supplementary file 6 — Source Data for Figure 5 [file MSB-17-e10105-s004.zip › Figure5A_sourcedata/GSEA_3017/hallmarks_stateC.GseaPreranked.1621934520804/HALLMARK_HYPOXIA.html]

Details for gene set HALLMARK\_HYPOXIA[GSEA]

|  || Dataset | state13017 |
| Phenotype | NoPhenotypeAvailable |
| Upregulated in class | na\_neg |
| GeneSet | HALLMARK\_HYPOXIA |
| Enrichment Score (ES) | -0.41917023 |
| Normalized Enrichment Score (NES) | -2.0156865 |
| Nominal p-value | 0.0015503876 |
| FDR q-value | 0.0066035753 |
| FWER p-Value | 0.029 |
Table: GSEA Results Summary

  

Fig 1: Enrichment plot: HALLMARK\_HYPOXIA      
 Profile of the Running ES Score & Positions of GeneSet Members on the Rank Ordered List

  

| PROBE | GENE SYMBOL | GENE\_TITLE | RANK IN GENE LIST | RANK METRIC SCORE | RUNNING ES | CORE ENRICHMENT || 1 | NEDD4L |  |  | 47 | 0.435 | -0.0423 | No |
| 2 | CSRP2 |  |  | 72 | 0.376 | -0.0495 | No |
| 3 | STC1 |  |  | 210 | -0.255 | -0.2666 | No |
| 4 | TPST2 |  |  | 226 | -0.259 | -0.2690 | No |
| 5 | DTNA |  |  | 281 | -0.271 | -0.3387 | No |
| 6 | SERPINE1 |  |  | 323 | -0.286 | -0.3843 | No |
| 7 | PPP1R15A |  |  | 342 | -0.293 | -0.3888 | No |
| 8 | CHST2 |  |  | 350 | -0.297 | -0.3735 | No |
| 9 | FOS |  |  | 377 | -0.309 | -0.3905 | Yes |
| 10 | SLC2A1 |  |  | 383 | -0.311 | -0.3705 | Yes |
| 11 | CHST3 |  |  | 389 | -0.314 | -0.3502 | Yes |
| 12 | S100A4 |  |  | 395 | -0.318 | -0.3294 | Yes |
| 13 | SLC2A3 |  |  | 422 | -0.336 | -0.3440 | Yes |
| 14 | KLF6 |  |  | 429 | -0.339 | -0.3231 | Yes |
| 15 | VEGFA |  |  | 436 | -0.345 | -0.3016 | Yes |
| 16 | MIF |  |  | 442 | -0.347 | -0.2782 | Yes |
| 17 | HSPA5 |  |  | 447 | -0.350 | -0.2528 | Yes |
| 18 | BCAN |  |  | 449 | -0.351 | -0.2219 | Yes |
| 19 | CAVIN1 |  |  | 481 | -0.387 | -0.2405 | Yes |
| 20 | LDHA |  |  | 498 | -0.412 | -0.2304 | Yes |
| 21 | JUN |  |  | 503 | -0.425 | -0.1980 | Yes |
| 22 | F3 |  |  | 505 | -0.432 | -0.1596 | Yes |
| 23 | IRS2 |  |  | 516 | -0.444 | -0.1360 | Yes |
| 24 | IGFBP3 |  |  | 538 | -0.493 | -0.1272 | Yes |
| 25 | GPC1 |  |  | 555 | -0.536 | -0.1056 | Yes |
| 26 | ANXA2 |  |  | 563 | -0.567 | -0.0653 | Yes |
| 27 | MT2A |  |  | 566 | -0.575 | -0.0155 | Yes |
| 28 | CA12 |  |  | 586 | -0.717 | 0.0176 | Yes |
Table: GSEA details [plain text format]

  

Fig 2: HALLMARK\_HYPOXIA: Random ES distribution      
 Gene set null distribution of ES for **HALLMARK\_HYPOXIA**
